# The Role of Multidetector CT in the Diagnosis of Retroperitoneal Fibrosis: Report of a Case

**DOI:** 10.5812/iranjradiol.6343

**Published:** 2012-03-25

**Authors:** Hossein Ghanaati, Mehdi Mohammadifar, Mahsa Ghajarzadeh, Kavous Firouznia, Marzieh Motevalli, Amir Hossein Jalali

**Affiliations:** 1 Department of Radiology, Medical Imaging Center, Advanced Diagnostic and Interventional Radiology Research Center (ADIR), Imam Khomeini Hospital, Tehran University of Medical Sciences, Tehran, Iran; 2 Brain and Spinal Injury Research Center (BASIR), Tehran University of Medical Sciences, Tehran, Iran; 3 Department of Radiology, Shahid Radjaee Cardiovascular Medical Center, Tehran, Iran

**Keywords:** Multidetector Computed Tomography, Retroperitoneal Fibrosis, Diagnosis, Abdominal Pain

## Abstract

Herein, we report a 40-year old man who presented with flank and abdominal pain with dilatation of the bilateral pyelocalyceal system detected in ultrasonography. Computed Tomography (CT) scan showed a soft tissue mass at the level of the fourth and fifth lumbar vertebrae in the retroperitoneal region. There were no blood flow signals in 64-slice multidetector CT (MDCT) which confirms the Retroperitoneal Fibrosis (RPF). Pathological examination showed infiltration of plasma cells, macrophages, lymphocytes and eosinophils accompanied by fibrosis, which is consistent with idiopathic RPF. In conclusion, 64-slice MDCT imaging is useful in the diagnosis of RPF.

## 1. Introduction

Retroperitoneal fibrosis (RPF) is a rare inflammatory disease (incidence: 1:200,000 to 1:500,000), which can be idiopathic or secondary. This condition may present as low back or/and abdominal pain with or without flank tenderness [1]. Atherosclerosis, drugs, radiation, infections and surgery have important roles in the development of RPF [2]. Immunological response to antigens generated from the atheromatous plaques, soluble lipids and oxidized low-density lipoproteins near the periaortic tissues may lead to RPF [3]. Chronic infectious diseases, malignancies and drug consumption should be excluded to confirm idiopathic RPF diagnosis [1].

Computed Topography CT scan and Magnetic Resonance Imaging (MRI) are important in the diagnosis of RPF [1]. MDCT as well as MRI have been known in deter mining the extension and situation of RPF extension and the situation of ureters and vessels near the fibrotic mass [4]-[6]. Contrast-enhancement of the soft tissues around the infrarenal aorta or iliac vessels without aneurysmal evidence of the infrarenal aorta in CT or MRI and no blood signals in 64-channel multi-detector row CT (MDCT-64) are indicatives of RPF [4]. The aim of this study is to report a rare case of RPF which was diagnosed by MDCT.

## 2. Case Presentation

A 40-year-old man was referred to Imam Khomeini Hospital in September 2007 with pain in the flank and abdomen and also flank tenderness. He had a negative medical history, no history of radiation exposure and no previous surgery or suspicious infectious diseases.

Laboratory findings; namely, the complete blood count (CBC), erythrocyte sedimentation rate (ESR) and renal function tests were normal. Ultrasound showed bilateral dilatation of the pyelocalyceal system with normal renal parenchymal thickness.

**Figure 1:**
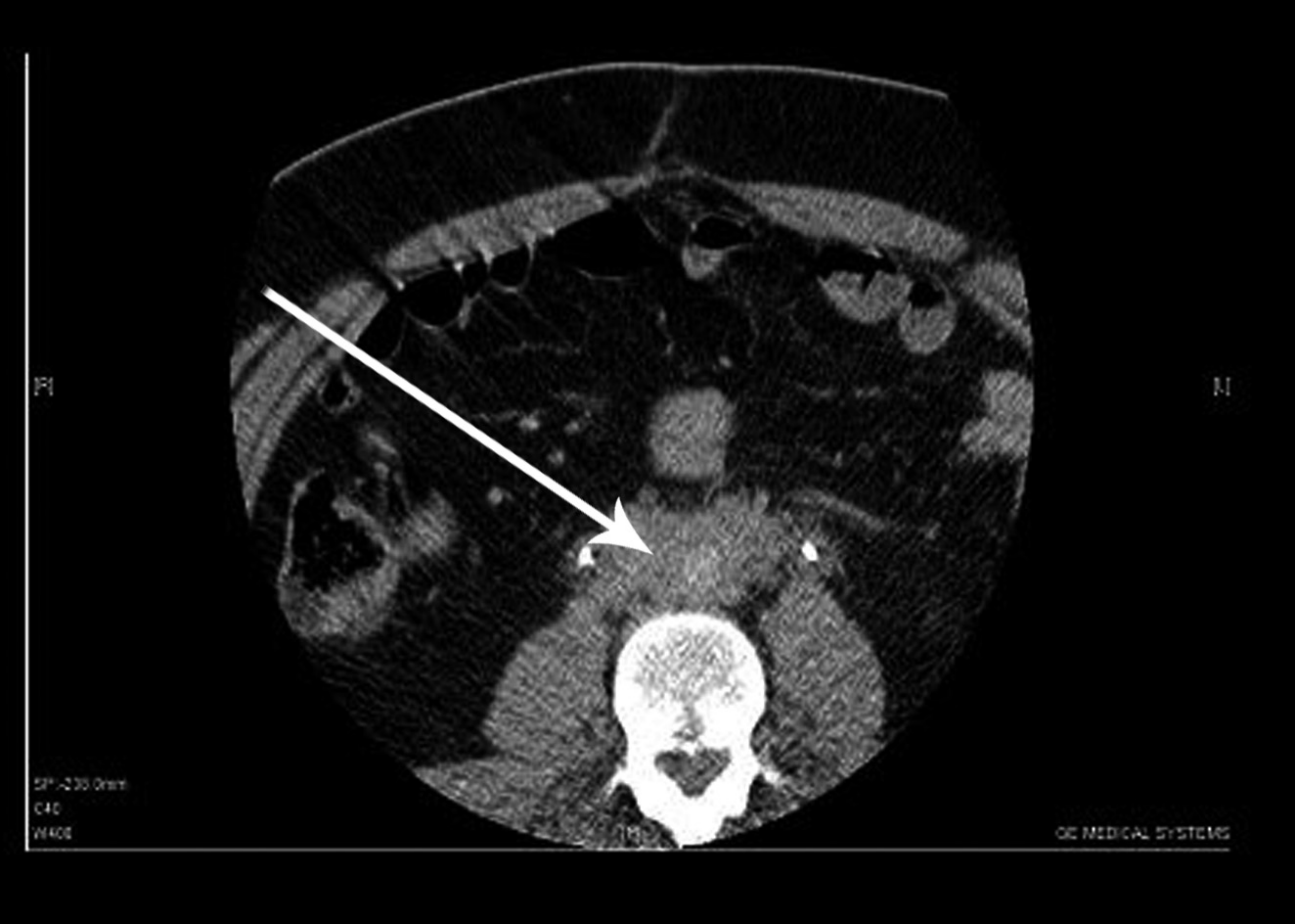
A 40-year-old man presenting with pain in the flank and abdomen together with flank tenderness CT scan without contrast shows a soft tissue mass (arrow) which is located in the retroperitoneal area at the level of the fourth and fifth lumbar vertebrae.

Conventional incremental CT scan (Toshiba X Vision, Tokyo, Japan) demonstrated a mass of soft tissue which was located in the retroperitoneal area at the level of the fourth and fifth lumbar vertebrae (Figure 1).

**Figure 2:**
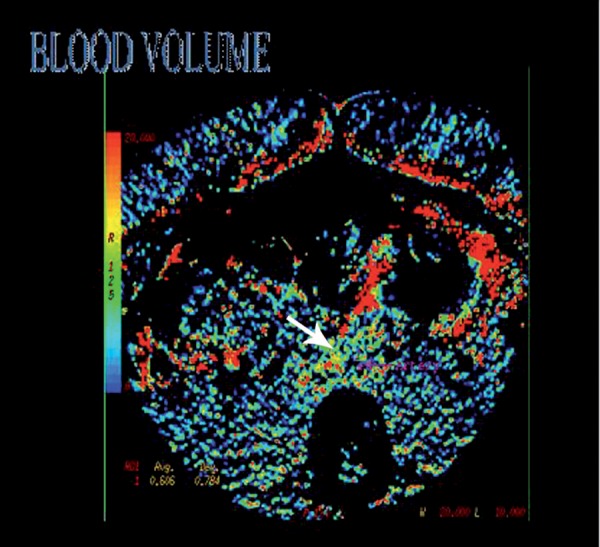
Perfusion CT scan with MDCT 64 shows the retroperitoneal mass (arrow) without blood flow signals.

Bilateral hydronephrosis and midline shift of the ureters were detected. For differentiating idiopathic RPF from malignancies or other retroperitoneal masses, perfusion CT scan with MDCT 64 (GE 64 Slice, Milwaukee, USA) (slice thickness: 0.6 mm, kVp: 120, mAs: 450-600, Rotation time: 0.7 s) was performed, in which no blood flow signals were detected in the mass (Figure 2).

Needle biopsy under CT scan guidance was performed for confirmation of the RPF. Pathological examination discovered infiltration by plasma cells, macrophages, lymphocytes and eosinophils with fibrosis, consistent with idiopathic RPF. The patient was treated by prednisone 1 mg/kg/day for one month and then gradually tapered to a maintenance dose of 5 mg/day for two years. After 3 months of therapy, the patient was generally well.

## 3. Discussion

RPF is a rare inflammatory disease without an obvious etiology and hard to diagnose because of its non-specific symptoms.

More than 25% of RPF cases (similar to this case) have normal laboratory findings [7]. Resembling previous studies, abdominal and flank pain were the presenting symptoms in our case and the mass was located near the infrarenal aorta and iliac arteries. In a previous report, Gluhovschi et al. reported a case of a 45-year-old woman who presented with diffuse lumbar pain and fever. Unlike our case, she used several drugs such as desmopressin, perindopril and indapamid. She had been treated for urinary infections several times. During the evaluation, she had a creatinine level of 1.74 mg/dL and ESR of 46 mm/1h. MDCT showed a dense tissue mass along the distal portion of the thoracic and the abdominal aorta and thickening of both kidney cortices and hydrocalycosis [8].

Immediate diagnosis of RPF is vital for preserving renal function, avoidance of hydroceles or edema due to obstruction or compression of the retroperitoneal vessels and vena cava [9]. Differentiating from conditions that mimic RPF symptoms such as retroperitoneal abscess, infections and malignancies is important for appropriate treatment. Although ultrasonography is the first diagnostic modality, it is not the best imaging modality for assessing RPF. Uni-or bilateral hydronephrosis, a hypoechoic and well-defined mass are characteristics of RPF in ultrasonography. Assessing the periaortic region may be difficult, particularly in obese cases and unfilled bowel loops by gas or fluid that may result in misdiagnosis.

MDCT is a perfusion assessment modality used to assess patients with nonspecific abdominal pain. Using MDCT, enormous images can be afforded and more data and better measurement of vascularity and perfusion may be possible [6]. It depicts complete images of the abdomen and retroperitoneum, free of overlying structures. It also provides accurate multiplanar reformations; three dimensional reconstruction and surface rendering that are helpful for demonstrating the extent of the disease and providing detailed information of complications. Extension of fibrotic masses and periaortic area involvement can be detected easily by MDCT [10][11]. It is a non-invasive modality used for qualitative and quantitative information on the topic of physiologic parameters such as vascularity. Besides, it has an important role in monitoring the management of tumors after chemotherapy and radiation therapy.

There are three types of CT perfusion protocols according to the number of images, injection rate, slice thickness, image frequency, acquisition and analysis method. Thinner slices, high scan frequency and lower tube current (mAs) are characteristics of protocol 1; while protocol 2 has lower noise, higher mAs, greater slice thickness and lower imaging frequency. Low image frequency, higher tube current and greater slice thickness are expected by means of protocol 3. In this way, tissue perfusion or blood volume measurements can be achieved easily and assessment of the vascular permeability will be possible [6].

High iodine concentration agent (370-400 mg/L) is recommended for CT-perfusion, due to its greater tissue enhancement and better perfusion quantification. Contrast media (40-50 mL) with a high injection rate (4-6 mL/s) followed by 40 mL saline at a high injection rate (4-6 mL/s) is recommended for CT-perfusion [12].

After injection of contrast media, temporal changes in contrast enhancement along with time-attenuation curves displacement will be analyzed to show the vascularity of the tissue. During the first 40-60 s, contrast media goes through vessels. During 45-60 s, a rapid series of images should be captured to evaluate perfusion and blood volume, which can show increased micro-vessel density (MVD). By passage of the contrast media into the extravascular space, the second phase starts and vascular permeability may be evaluated [6].

Qualitative investigation are carried out with color maps generated by software for each perfusion parameter. Numerical values calculated for each pixel of CT-image are indicatives of specific colors established by the operator to distinguish areas with different perfusions. In this case, yellow and red are indicatives of high numerical values (higher perfusion), while green and blue are representatives of low numerical values (low perfusion).

MDCT has been known as useful as MRI for RPF evaluation, but intravenous (IV) contrast application may be harmful in high risk cases due to nephrotoxicity, while differentiation between vessels and lymph nodes and inflammatory processes will be possible [13]. In our case, the mass did not reveal blood flow signals.

In comparison to 16-channel MDCT systems, 64-channel MDCT systems led to further increased spatial resolution (0.4 mm isotropic voxels) and gantry rotation times as low as 0.33 second, while 16-channel MDCT has gantry rotation times as low as 0.375 second ([14]-[16]). This method improves image quality leading to a better diagnosis.

Contrast-enhancement of soft tissues around the infrarenal aorta or iliac vessels without aneurysmal evidence of the infrarenal aorta in CT scan and no blood signals in 64-channel MDCT are diagnostic for RPF [3]. By this modality, differentiation between RPF and other retroperitoneal masses can be determined carefully.

In conclusion, 64 slice CT imaging is useful in the diagnosis of RPF.

## References

[1] Vaglio A, Salvarani C, Buzio C (2006). Retroperitoneal fibrosis. Lancet.

[2] Scheel PJ, Feeley N (2009). Retroperitoneal fibrosis: the clinical, laboratory, and radiographic presentation. Medicine (Baltimore)..

[3] Vaglio A, Greco P, Corradi D, Palmisano A, Martorana D, Ronda N (2006). Autoimmune aspects of chronic periaortitis. Autoimmun Rev.

[4] Smith RA, Cokkinides V, Brawley OW (2009). Cancer screening in the United States, 2009: a review of current American Cancer Society guidelines and issues in cancer screening. CA Cancer J Clin.

[5] Kottra JJ, Dunnick NR (1996). Retroperitoneal fibrosis. Radiol Clin North Am.

[6] Miles KA, Griffiths MR, Comber L, Keith CJ, Fuentes M (2002). Functional imaging of cancer: combining perfusion CT with FDG-PET. Cancer Imaging.

[7] Gilkeson GS, Allen NB (1996). Retroperitoneal fibrosis. A true connective tissue disease. Rheum Dis Clin North Am.

[8] Gluhovschi G, Bozdog G, Miclaus G, Puscasiu T, Gluhovschi C, Bob F (2011). Idiopathic retroperitoneal fibrosis with particular perirenal and intrarenal extension associated with left renal artery stenosis. The atheromatous periaortitis with retroperitoneal fibrosis suggests a pathogenic relationship between atherosclerosis and fibrosis?. Wien Klin Wochenschr.

[9] Duffy TP (1994). Clinical problem-solving. An anatomy lesson. N Engl J Med.

[10] Higgins PM, Aber GM (1990). Idiopathic retroperitoneal fibrosis--an update. Dig Dis.

[11] Hollerweger A (2007). Colonic diseases: the value of US examination. Eur J Radiol.

[12] Petralia G, Preda L, D’Andrea G, Viotti S, Bonello L, De Filippi R (2010). CT perfusion in solid-body tumours. Part I: Technical issues. Radiol Med.

[13] Moroni G, Dore R, Collini P (2005). Idiopathic retroperitoneal fibrosis. J Nephrol.

[14] Flohr T, Ohnesorge B, Bruder H, Stierstorfer K, Simon J, Suess C (2003). Image reconstruction and performance evaluation for ECG-gated spiral scanning with a 16-slice CT system. Med Phys.

[15] Flohr TG, Schoepf UJ, Kuettner A, Halliburton S, Bruder H, Suess C (2003). Advances in cardiac imaging with 16-section CT systems. Acad Radiol.

[16] Flohr TG, Stierstorfer K, Ulzheimer S, Bruder H, Primak AN, Mc-Collough CH (2005). Image reconstruction and image quality evaluation for a 64-slice CT scanner with z-flying focal spot. Med Phys.

